# Pediatric Hypertension: A Condition That Matters

**DOI:** 10.3390/children11050518

**Published:** 2024-04-26

**Authors:** Martina Avesani, Giuseppe Calcaterra, Jolanda Sabatino, Giulia Pelaia, Irene Cattapan, Francesco Barillà, Francesco Martino, Roberto Pedrinelli, Pier Paolo Bassareo, Giovanni Di Salvo

**Affiliations:** 1 Division of Pediatric Cardiology, Department for Women’s and Children’s Health, University of Padua, 35128 Padua, Italy; martina.avesani@aopd.veneto.it (M.A.); irene.cattapan@aopd.veneto.it (I.C.); 2 Post Graduate Medical School, University of Palermo, 90128 Palermo, Italy; peppinocal7@gmail.com; 3 Department of Experimental and Clinical Medicine, Magna Graecia University of Catanzaro, 88100 Catanzaro, Italy; sabatino@unicz.it (J.S.); giulia.pelaia@studenti.unicz.it (G.P.); 4 Department of Systems Medicine, Tor Vergata University, 00133 Rome, Italy; francesco.barilla@uniroma2.it; 5 Department of Internal Medicine, Anaesthesiology, and Cardiovascular Sciences, Sapienza University, 00185 Rome, Italy; francesco.martino46@libero.it; 6 Department of Surgical, Medical and Molecular Pathology and Critical Care Medicine, University of Pisa, 56126 Pisa, Italy; roberto.pedrinelli@unipi.it; 7 School of Medicine, University College of Dublin, Mater Misericordiae University Hospital, D07 KH4C Dublin, Ireland; piercard@inwind.it

**Keywords:** pediatric cardiology, hypertension, treatment

## Abstract

Systemic hypertension has been considered mainly as an adult health issue for a long time, but it is now being increasingly acknowledged as a significant problem also among pediatric patients. The frequency of pediatric hypertension has grown mostly because of increases in childhood obesity and sedentary lifestyles, but secondary forms of hypertension play a role as well. Considering that unaddressed hypertension during childhood can result in enduring cardiovascular complications, timely identification and intervention are essential. Strategies for addressing this disease encompass not only lifestyle adjustments, but also the use of medications when needed. Lifestyle modifications entail encouraging a nutritious diet, consistent physical activity, and the maintenance of a healthy weight. Moreover, educating both children and their caregivers about monitoring blood pressure at home can aid in long-term management. Thus, the aim of this review is to discuss the etiologies, classification, and principles of the treatment of hypertension in pediatric patients.

## 1. Introduction

Around 3.5–5% of children in Western countries experience primary or secondary hypertension (HTN) [1]. The rates of HTN tend to be elevated in those with specific chronic conditions like obesity and diabetes, where prevalence ranges from 3.8% to 24.8% and 6% to 16%, respectively [2,3].

Addressing HTN in children and adolescents is crucial due to the fact that a high blood pressure (BP) in childhood may be reflected later in adulthood [3]. Furthermore, evidence suggests that the onset of HTN in childhood is linked to major adverse cardiovascular events such as atrial fibrillation, cerebral ischemia, myocardial infarction, and renal dysfunction later in adulthood [2,3,4,5]. Thus, it is mandatory to promptly recognize this disease in the pediatric population and promptly treat it.

This article summarizes the classification of HTN and its diagnostic and therapeutic algorithm, aiming to aid practitioners in the effective management of HTN in children and adolescents.

## 2. Etiologies

HTN can be defined as primary or secondary.

### 2.1. Primary Hypertension

In Western countries, primary hypertension has become the prevailing diagnosis among children (aged >6 years) and adolescents. These patients are usually overweight or obese and have a positive familial history of HTN. In this form, HTN is mostly asymptomatic and related to increased systolic values [6,7]. However, a study conducted by Ding et al. showed that nearly 50% of pediatric patients with traditional risk factors for essential HTN were, in the end, diagnosed with a secondary form, so adequate screening is mandatory [8].

### 2.2. Secondary Hypertension

Forms of secondary HTN must be promptly recognized to address the underlying cause and prevent disease in the target organ. Children suffering from HTN determined by organic causes account nowadays for half of all hypertension diagnoses [2].

Overall, secondary HTN begins earlier and shows worse patterns than the primary form, with a trend of above-normal diastolic BP values during the day and higher systolic values during the night [9].

Some specific congenital heart diseases (CHDs) are, by definition, at risk of HTN [10]. Among these, it is well known that coarctation of the aorta (COA) is strictly associated with HTN. Studies have revealed that around 30% of pediatric patients develop HTN post-early COA repair, with prevalence rising to 68% in adults [11,12,13]. The etiology of HTN in COA is multifaceted, involving increased aortic stiffness and reduced distensibility, often persisting post-repair. Histological analyses have revealed abnormalities in the smooth muscle, elastic fibers, and collagen within the aortic wall of COA patients. Additionally, specific aortic arch morphologies, such as gothic arch geometries, and certain surgical techniques may exacerbate HTN. Postoperative complications like residual narrowing or re-coarctation can be also a hidden etiology of HTN in this specific population. Neurohormonal shifts, including stimulation of the renin–angiotensin–aldosterone system and alterations in baroreceptors, contribute significantly to HTN progression in COA. Despite successful repair, individuals with COA face lower survival rates compared to the general population, primarily due to accelerated atherosclerosis [11,12,13,14,15].

Beyond COA, various other CHDs also predispose individuals to HTN, including Tetralogy of Fallot, D-transposition of great arteries, common arterial trunk, single ventricles with Fontan palliation, and some syndromes like Williams Syndrome and Turner Syndrome [16,17,18,19,20,21,22]. For instance, renal stenosis, but also supravalvular aortic stenosis, typical features of Williams Syndrome, may cause HTN [20]. Also, an early impairment in aortic wall elasticity is observed in children with bicuspid aortic valve (BAV), increasing the risk of HTN. Finally, Turner Syndrome, characterized by several cardiovascular abnormalities, is also linked to a higher prevalence of HTN, particularly nocturnal HTN [21,22].

Apart from specific CHDs, shared characteristics among CHDs can predispose individuals to chronic kidney injury and subsequent HTN. Indeed, chronic hypoxia in cyanotic CHDs stimulates erythropoietin production, leading to erythrocytosis and increased blood viscosity [23,24]. This results in glomerular hypertension and chronic nephropathological damage, such as glomerulosclerosis. Cardiac catheterization or surgery itself can cause disturbances in cardiac receptors, enhancing sympathetic activity and potentially resulting in acute and chronic kidney damage, particularly in patients with complex CHDs.

The persistent elevation of important regulators of renal physiology, such as natriuretic peptides, renin, aldosterone, and norepinephrine, is observed in children with CHDs, even years after surgical correction. Additionally, chronic volume overload and the employment of medications, such as diuretics and angiotensin-converting enzyme inhibitors, can promote glomerulosclerosis and chronic kidney disease in long-term follow-ups [23,24].

On the other hand, a sedentary lifestyle with scarce or insufficient physical activity may increase the cardiovascular risk in children with CHDs, leading, in turn, to HTN. Obesity is linked to worse cardiac remodeling and function in children with COA, even after a successful surgical or percutaneous correction. Nevertheless, despite being widely acknowledged as crucial, various obstacles impede the encouragement of a healthy lifestyle in these patients. These include excessive parental protection, insufficient patient education, and a lack of specialized rehabilitation plans.

Finally, physicians should consider acquired modifiable risk factors and investigate other secondary causes of HTN, even in patients with CHDs.

Indeed, up to school age, chronic renal disorders are in second place as the most common cause of secondary HTN, whereas kidney parenchymal diseases represent the main culprits in children aged between 6 and 10 years [25]. In a large single-center study including 1025 subjects with secondary HTN, nephrological issues accounted for 68% of all cases, with a prevalence of renal scarring of around 50% and chronic glomerulonephritis of around 20% among the included subjects. Less-represented causes like hydronephrosis, renal polycystic disease, and acute renal failure due to hemolytic uremic syndrome accounted for the rest of the cases [26]. Renovascular HTN due to renal arterial stenosis is a rather uncommon but not negligible determinant of HTN, which frequently leads to diagnosis delay. Its underlying etiology shows different distributions following ethnicity: in Europe, fibromuscular dysplasia constitutes the most common lesion, while, in Asia, Takayasu Arteritis represents the predominant etiology, and Moya Moya accounts for most cases in Korea [27,28]. In all these conditions, vessels other than renal arteries are often affected, implying the necessity to broaden the diagnosis process to other organs.

Endocrinologically based secondary hypertension is less frequent in children and accounts for 5–10% of all causes [29]. Therefore, screening for endocrine hypertension in children should occur only following the exclusion of renal and cardiovascular causes [29]. Hypertension due to endocrinological issues is frequently part of a syndromic pattern, where some other clinical signs such as pubertal delay, growth restriction, obesity, or features suggestive of Cushing’s disorder might be recognized. Among all endocrinological causes of HTN, pathologies that lead to low renin levels and high mineralocorticoids levels (i.e., Cushing’s syndrome, Gordon syndrome, Liddle syndrome, generalized glucocorticoid resistance, adrenal steroid synthetic defects, and apparent mineralocorticoid excess) are the most frequent [30]. Mineralocorticoids, in fact, stimulate sodium retention, HTN, potassium elimination, fluid accumulation, and renin suppression. Furthermore, pheochromocytoma is gaining attention nowadays as a possible cause of HTN, since the advancement of molecular genetics showed that it can be familiar in up to 25% of cases. Children with pheochromocytoma often exhibit sustained HTN between the ages of 6 and 14 years, although approximately 10% may also present as normotensive, and orthostatic hypotension is also relatively frequent.

Moreover, Polycystic Ovarian Syndrome (PCOS) must be mentioned as well, since hyperinsulinemia, hyperandrogenism, and increased sympathetic activity might increase the risk of HTN in these patients, also through weight gain and obesity development.

Finally, it is important to remember that HTN can also be the secondary effect of pharmacological treatments such as corticosteroids, the chronic use of NSAIDs, contraceptive pills, ADHD medications, and environmental factors like lead exposure [31]. Of note, diastolic BP elevation seems to be more predictive of secondary HTN [32].

## 3. Definition and Classification

American and European guidelines have provided different definitions and cut-offs for elevated blood pressure and stages of hypertension in pediatric patients over time. The consensus document recently published by the European Society of Cardiology (ESC) determined that the definition of HTN should align with the guidelines proposed by the American Academy of Pediatrics for individuals aged 16 and under [33]. These guidelines use new percentile tables derived from a reference population excluding youths with overweight/obesity [2].

Based on these recommendations, blood pressure is categorized as elevated if the systolic and/or diastolic readings exceed the 95th percentile in three distinct measurements for patients aged <13 years. Furthermore, HTN is further classified into stages: first-stage HTN accounts for pressure values ≥95th percentile, while second-stage HTN is defined as pressure values ≥95th percentile + 12 mmHg. For patients above 13 years, a streamlined blood pressure categorization in line with the ACC/AHA adult BP guidelines was adopted. This approach uses a threshold of 120/80 mmHg to identify an elevated BP, irrespective of gender. Lastly, for those who are 16 years or older, the endorsed threshold is set at 130/85 mmHg [1,2,33].

**Figure 1:**
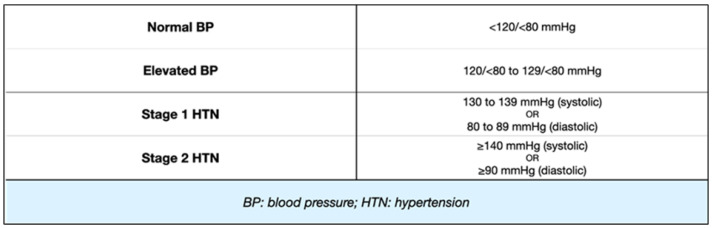
Classification of BP/HTN in children aged ≥13 years. Cut-off values for BP in children ≥13 years of age. Four categories are identified, based on absolute values of systolic or diastolic pressure.

**Figure 2:**
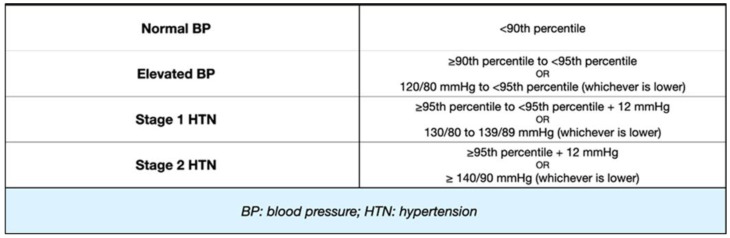
Classification of BP/HTN in children aged between 1 and 13 years. Cut-off values for BP in children <13 years of age using percentiles or absolute values.

These recommendations are summarized in Figure 1 and Figure 2.

Although arterial HTN seems to be a condition mainly affecting children aged 7 years and older and adolescents, recently, the awareness towards this condition has increased also in neonatal age. For an extended period, this illness has been overlooked because of imprecise measurement methods, exacerbated by significant fluctuations in typical reference values. These values encompass factors like gestational age, postnatal age, birth weight, and gender [34]. Clinical information and guidelines for managing this condition are not yet available, however, we can indicate which are the fundamental items for rapid recognition.

Providing a univocal definition of neonatal HTN is very complex, but it is possible to define it as a BP value that exceeds the 95th percentile by correlating three essential variables: post-menstrual age, gestational age, and birth weight [35].

Recent studies established that, in preterm infants, the incidence of HTN is more elevated than in term babies. Also, during the first days of life, in preterm newborns, a rapid increase in blood pressure occurs compared to full-term children, in whom it resolves earlier, within 3–4 days [36]. This is because preterm infants have more risk factors, such as bronchopulmonary dysplasia, cardiac diseases, acute kidney damage, the utilization of umbilical arterial catheters, being underweight at birth, neonatal opioid withdrawal syndrome, and the use of neonatal ECMO. Therefore, prematurity itself represents a risk factor for the onset of HTN [37].

To perform reliable measurements, the newborn must be resting or sleeping, since crying, feeding, and states of agitation can interfere, causing increases in the recorded values [38]. Invasive and non-invasive methods can be used: intra-arterial measurement is the most accurate and consists of the catheterization of a large caliber artery; non-invasive methods provide the oscillometry technique using an appropriate cuff, proportional to the length of the limb.

A reason why neonatal HTN is underdiagnosed is that the neonate is often asymptomatic, and it is rare to notice signs like congestive cardiovascular disease, food refusal, or lack of growth [39]. However, it is possible that the finding of HTN is associated with cardiac, respiratory, neurologic, and, especially, renal signs and symptoms. Its etiology is profoundly different from the childhood form and it mainly involves renal causes, as evaluated in the well-known Assessment of Worldwide Acute Kidney Injury Epidemiology in Neonates (AWAKEN) study [40]. Kidney diseases related to increased blood pressure can either arise at birth or appear later. Congenital conditions include polycystic kidney disease (PKD), multicystic dysplastic kidney disease (MCDK), and congenital ureteropelvic junction disfunction. Among acquired kidney diseases, acute tubular necrosis is one of the most frequent, often related to sepsis or perinatal asphyxia and nephrocalcinosis. Umbilical arterial catheter (UAC)-associated thrombosis is another potential cause. The UAC placement can lead to the formation of thrombi, which can cause the partial or complete occlusion of the abdominal aorta, resulting in renal hypoperfusion; these thrombi can also embolize in the renal vascular network, causing renal infarctions and increasing renin release [41]. Causes that should not be underestimated in neonatal age are bronchopulmonary dysplasia and iatrogenic causes such as fluid overload and medications (glucocorticoids). In most cases, treating the correctable disorders helps to resolve the condition, however, if it remains above the 99th percentile, medical therapy is suggested [42].

## 4. Screening for HTN

As reported by the largest professional association of pediatricians in the United States (APP), BP must be assessed by placing the cuff at the biceps, aligned with the heart. The cuff’s linear measure needs to encompass about the 90% of the upper limb’s diameter, and the width should be about 50%. The child should be in a silent area for more or less than 5 min in preparation for blood pressure measurement, and it is important that they can stay seated with proper back support and uncrossed legs.

If the first value detected exceeds the 90th percentile, healthcare has to conduct two more BP measurements using either oscillometric or auscultatory methods during the same visit, and then calculate the average of these three measurements. If auscultation is used, the mean value allows for categorizing the child’s BP range. Conversely, if the intermediate value also falls into the 90th percentile or higher, two auscultatory measurements must be detected and averaged to establish the blood pressure class; this is necessary, since the oscillometer easily risks overestimating systolic and diastolic BP measurements in comparison to the auscultatory method [2,43].

In regard to the recurrence of BP surveillance in children and teenagers, there is no clear recommendation. Indeed, according to the 2020 U.S. Preventive Services Task Force (USPTF), there is not sufficient evidence to suggest for or against monitoring for elevated BP in children and teenagers in the age group from 3 to 18 years who do not show signs of hypertension [44]. By contrast, AAP guidelines recommend measuring BP at wellbeing child visits for children ≥3 years annually in otherwise healthy children. More frequent BP measurements are recommended in the following clinical scenarios: obesity, mellitus diabetes, renal diseases, coarctation of the aorta, and the assumption of medications that increase BP [2].

## 5. Diagnostic Workup

Once a diagnosis of HTN is made, it is essential to determine the form of hypertension (primary or secondary) and to assess potential organ damage that, in pediatric patients, includes mainly the heart and kidneys.

The first step in the diagnostic workup is the patient’s family and personal history, including perinatal factors (i.e., maternal HTN, low birth weight, and preterm), nutritional data, including the amounts of fruits and vegetables consumed and potential high intake of sodium, their level of physical activity, and psycosocial factors (i.e., stress, depression, or bullying) [45,46,47,48].

Secondly, a physical examination including anthropometric parameters such as weight, height, their relative percentiles, and BMI is essential. Also, various body systems should be carefully assessed to identify potential causes of secondary HTN. For example, cardiac and chest auscultation and the assessment of femoral pulses may raise the suspicion of COA, while the presence of obesity associated with a moon face, acne, and hirsutism could identify patients with Cushing syndrome.

Laboratory tests that include basic screening exams and additional specific tests based on clinical suspicion are also required. All patients should undergo blood tests including electrolytes, urea, creatinine, and lipid profile, as well as urinalysis. Additionally, in obese children and adolescent patients, hemoglobin A1c, liver function, and fasting lipid panels should be checked. Lastly, optional tests such as a complete blood count, an assessments of thyroid function and fasting serum glucose, as well as a drug screening, can be performed based on initial screening [49].

In children, currently, the evaluation of secondary forms of HTN and potential organ damage includes mainly:

1. Cardiac assessment: although it has been extensively used to screen for HTN, electrocardiography is not currently recommended to rule out left ventricular hypertrophy (LVH) because of its very low positive predictive value and scarce sensitivity [50,51]. By contrast, echocardiography is the main diagnostic technique both to diagnose COA and to measure left ventricular mass, and, as consequence, potential LVH.

LVH is strongly associated with cardiovascular outcomes in adults, thus, it is fundamental to properly identify pediatric patients at risk for future complications [52]. The echocardiographic definition of LVH includes a left ventricular mass higher than 115 g for boys and 95 g for girls, both divided per body surface area (BSA). LV wall thickness and systolic function should also be assessed [2]. If heart damage is confirmed, echocardiography could be repeated after the initiation of hypertensive treatment to look for improvements in or worsening of the injury at intervals ranging from 6 to 12 months. In the case of absent heart damage, echocardiography may be repeated annually in patients with chronic stage 1 HTN that is incompletely treated, and in those with secondary forms or stage 2 HTN.

2. Renovascular evaluation: Doppler renal scans could be performed in children with abnormal urinalysis, renal function, or with HTN and hypokalemia to identify renal artery stenoses, especially in cooperative children aged >8 years and non-obese subjects [53]. Computed tomographic or magnetic resonance angiography may also be performed as an alternative in selected cases. The use of microalbuminuria in children as a marker of kidney damage is less established and is not currently recommended.
3. In children exhibiting low renin hypertension, hypokalemia, and with a family background of severe hypertension diagnosed during youth, refractory hypertension, cerebral vascular accidents, and heart failure causing death, genetic tests should be considered. Indeed, suspected monogenic forms such as Liddle’s syndrome, glucocorticoid-remediable aldosteronism, apparent mineralocorticoid excess, Gordon’s syndrome, mineralocorticoid receptor hypersensitivity syndrome, and hypertensive forms of congenital adrenal hyperplasia necessitate genetic analyses for accurate diagnosis in such cases [54].

## 6. Treatments of HTN

According to the AAP guidelines, an optimal BP level to achieve with treatments is <90th percentile or <130/80 mmHg [2]. Treatments can be differentiated into non-pharmacological and pharmacological, but it is important to remember that pharmacological options should always be accompanied by non-pharmacological treatments.

1. Non-pharmacological treatments: when and how to start.

In the case of elevated BP, the AAP guidelines suggest adopting preventive strategies such as lifestyle modification, including a healthy diet and the practice of physical activities. A patients’ family has an important role in these strategies, and should be involved in creating a healthy familial context, providing, for example, a balanced nutrition plan and a smoke-free environment, but advice could be sought from professional figures to manage nutrition and/or weight. BP should be checked by auscultation after 6 months and, if still elevated, after having excluded a gradient between upper and lower extremities, these strategies should be reinforced. If BP values are still high after 1 year, ambulatory BP monitoring (AMBP) and some screening tests should be performed as well [2].

The same lifestyle modifications and approach should be recommended in patients with a BP reading at stage 1, but BP should be checked initially within 1–2 weeks and, if persistent at the stage 1 level, within 3 months. At this point, AMBP and screening tests should be prescribed.

By contrast, in patients in whom the BP reading is consistent with stage 2, the above-mentioned strategies should be adopted as well, but BP values should be checked in 1 week or, as an alternative, patients can be referred to subspecialty care directly to investigate causes of secondary HTN. In the case where BP values are confirmed at stage 2 after 1 week, pharmacological treatment should be commenced. An admission to the Emergency Department is advisable for children who display symptoms or have a BP surpassing 30 mmHg of the 95th percentile (or >180/120 mm Hg in adolescents) [2].

As for lifestyle modifications, these include:

- Low consumption of sweets/sugar and a large consumption of fruits, vegetables, low-fat dairy products, whole grains, fish, poultry, nuts, and lean cuts of red meat. Lastly, sodium intake should be limited. [54,55].
- The practice of moderate to vigorous physical activity for 30–60 min at least 3 days/week. This practice should include mostly aerobic activities, but some resistance components are also allowed. Of note, in patients with stage 2 HTN, competitive sports should be avoided [33,56].
- The application of techniques such as awareness meditation and yoga for stress reduction [57].

After blood pressure is effectively managed, patients who are adhering to lifestyle modifications should have in-office appointments scheduled every 3 to 6 months [2].

2. Pharmacologic Treatment

For children who remain hypertensive despite lifestyle adjustments, those exhibiting symptoms with stage 2 HTN without easily modifiable factors like obesity, or any stage of HTN alongside diabetes or chronic kidney disease (CKD), initiating a medication treatment is recommended.

Overall, pharmacological treatment should begin with the minimum recommended dosage and switch to a different pharmacological class in the case where side effects appear. If no effect is obtained with the lowest recommended dose of a single drug, this can be increased up to full dosage. As an alternative, especially in patients with stage 2 HTN or organ damage, a low-dose combination can be started and increases up to the maximal dosage can be used in the case of inefficacy [33]. Of note, only a few studies testing combination therapies are available in children compared to adults; thus, further studies are needed [58].

Initial treatment choices comprise angiotensin-converting enzyme inhibitors (ACEi), angiotensin receptor blockers (ARBs), long-acting calcium channel blockers, and thiazide diuretics, but only ACEi and ARBs performed better than a placebo in decreasing systolic and diastolic BP, according to a meta-analysis published in 2018 [59].

Unless there is a specific contraindication, the typically preferred first-line medications when dealing with diabetes, proteinuria, and CKD are represented by ACE inhibitors and ARBs [2,33]. It should also be remembered that African-American children might need an increased initial dosage of ACE inhibitors or, as an alternative, a thiazide diuretic or long-acting calcium channel blocker can be started directly [60].

Because of undesirable side effects and considering that they have not demonstrated better outcomes when compared to other treatment options in adults, first-line therapy does not typically include beta-blockers, which are generally used in cases where the response to two or more of the preferred treatments is suboptimal [2].

**Figure 3:**
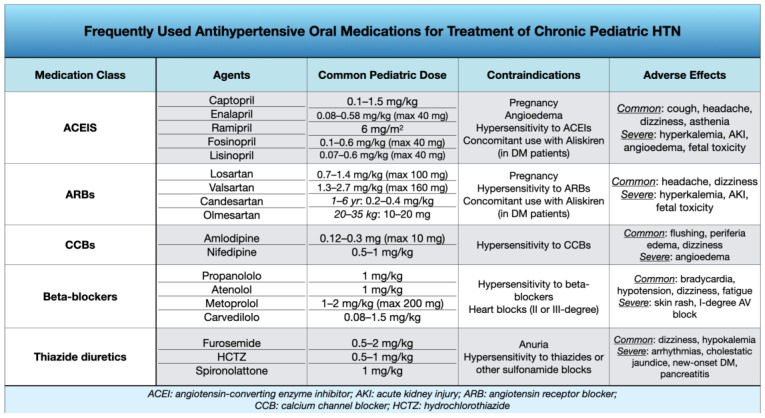
Pharmacologic treatment of Chronic Pediatric HTN. Main pharmacological classes, agents, dosages, contraindications, and side effects.

Figure 3 summarizes the most frequently used anti-hypertensive drugs in children and their dosages, side effects, and contraindications. (Figure 3).

Children and adolescents on pharmacological treatment should be monitored at the beginning every 4–6 weeks to check the efficacy and eventually increase the dosage and/or start an additional drug [2].

**Figure 4:**
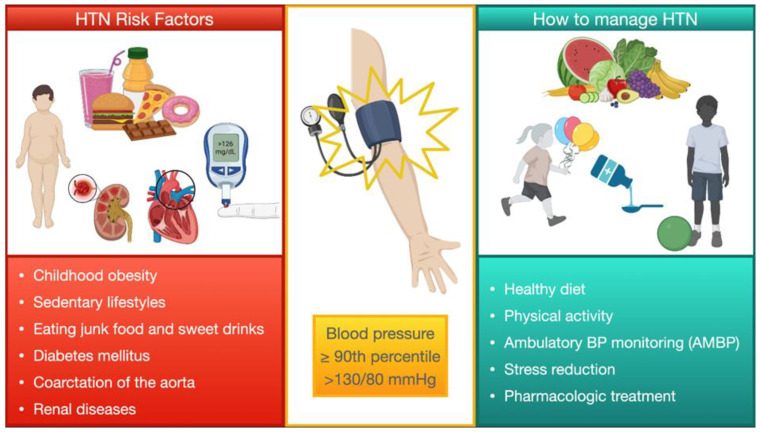
Summary of the general management of pediatric HTN. Management of HTN in children, including risk factors modifications, non-medical, and medical treatments.

After BP is effectively managed, follow-up appointments can be scheduled at intervals of every 3 to 4 months. It is also important to involve patients and their caregivers in BP monitoring, teaching them how to check BP at home and considering that home measurements appear to be more reproducible than those taken in a clinical setting [61]. All these strategies are summarized in Figure 4.

## 7. Conclusions and Future Perspectives

In conclusion, addressing HNT in children and adolescents requires accurate diagnoses, advising lifestyle adjustments, prescribing suitable first-line medications, and monitoring the effectiveness of these interventions.

Since overweight and obesity are becoming more and more frequent, primary HTN is becoming the prevailing diagnosis in Western countries. It is, however, important to consider and investigate causes of secondary HTN in the diagnostic process.

Considering that HTN can lead to significant cardiovascular events when not properly treated, a correct diagnosis, patient education, and treatments are mandatory, and these should be managed by expert professional figures. Similarly, the development of large clinical trials including only pediatric patients to test anti-hypertensive drugs would have a great impact on the management of HTN in this population.

## Data Availability

No new data were created or analyzed in this study. Data sharing is not applicable to this article.
